# Manganese dioxide-modified carbon paste electrode for voltammetric determination of riboflavin

**DOI:** 10.1007/s00604-016-1789-4

**Published:** 2016-02-29

**Authors:** Eda Mehmeti, Dalibor M. Stanković, Sudkate Chaiyo, Ľubomir Švorc, Kurt Kalcher

**Affiliations:** Institute of Chemistry, Analytical Chemistry, Karl-Franzens University, 8010 Graz, Austria; Department of Analytical Chemistry, Innovation Center of the Faculty of Chemistry, University of Belgrade, Studentski trg 12-16, Belgrade, 11000 Serbia; Electrochemistry and Optical Spectroscopy Research Unit, Department of Chemistry, Faculty of Science, Chulalongkorn University, 254 Phayathai Road, Pathumwan, Bangkok, 10330 Thailand; Institute of Analytical Chemistry, Faculty of Chemical and Food Technology, Slovak University of Technology in Bratislava, Radlinského 9, SK-812 37 Bratislava, Slovak Republic

**Keywords:** Differential pulse voltammetry, Cyclic voltammetry, Vitamin B_2_, Electrooxidation

## Abstract

A carbon paste electrode bulk was modified with MnO_2_ and investigated for use as an electrochemical sensor for riboflavin (vitamin B_2_) using differential pulse voltammetry (DPV). Riboflavin displays a well expressed oxidation peak at −0.15 V (versus Ag/AgCl) in solutions with a pH value of 2. Effects of pH value, pulse amplitude and pulse time were optimized by employing DPV. The signals obtained are linearly related to the concentrations of riboflavin in the range from 0.02 to 9 μM. Other features include a 15 nM detection limit, and good reproducibility (±3 %) and repeatability (±2 %). Interferences by common compounds were tested, and the method was successfully applied to the determination of riboflavin in pharmaceutical formulations where is gave recoveries in the range from 95 to 97 %.

**Graphical abstract:**
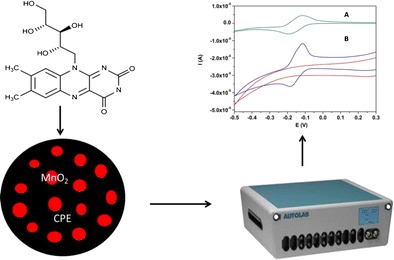
Manganese(IV) oxide was used as a modifier for the carbon paste electrode (MnO_2_/CPE) for improving its performance toward riboflavin oxidation. Cyclic voltammetry and differential voltammetry were used for characterization and determination of riboflavin, respectively.

Manganese(IV) oxide was used as a modifier for the carbon paste electrode (MnO_2_/CPE) for improving its performance toward riboflavin oxidation. Cyclic voltammetry and differential voltammetry were used for characterization and determination of riboflavin, respectively.

## Introduction

Vitamins are the important group of organic compounds and they are necessary for human health. They are required in the diet and to human body for normal growth and nutrition where their absence can lead to many diseases [1]. Riboflavin or Vitamin B_2_ is a water soluble vitamin and an essential component of flavoenzymes which plays a significant role in biochemical reactions of the human body [2]. It cannot be synthesized in human body therefore has to be obtained dietary from the sources such as liver, cheese, milk, meat, eggs, wines, and tea [3] therefore its insufficiency is associated with eye lesions and skin disorders [4].

Up to the date several analytical methods are used for the determination of Vitamin B_2_ such as HPLC [5] chemiluminescence [6], fluorescence [7] and capillary electrophoresis [8]. These methods provide highly sophisticated instrumentation setup and they are more time consuming [9]. Therefore, electrochemical methods have received great attention due to their simple, sensitive, low cost and rapid response time [10–12]. The use of chemically modified electrodes offers a tool for improving the performance of electrodes and several functional materials are used as a modifier for vitamin sensors [13, 14].

Carbon paste electrodes (CPE) are widely used as an electrode material for the development of various electrochemical sensors and biosensors and they can be simply modified [15]. The main advantages of carbon paste electrode are due to easy way of preparation, the new reproducible surface and low residual current in wide potential windows [16]. The modification of carbon paste electrodes with catalytic metals, has received also considerable attention [11]. There are already reported several carbon electrodes with numerous types of modifiers such as MnO_2_, Fe_3_O_4_, FeO, SnO_2_, CuO, Fe_2_O_3_ which are found to be very sensitive to acids and basis media, oxides of platinum group metals, complexes of copper, nickel, iron and chromium or/and also nanocomposites of these modifiers. The purpose of the use of these modifiers is due to the lowering overpotential for the oxidation or reduction of analytes, in comparison with unmodified electrodes. The obtained analytical responses are significantly higher and with high reproducibility of the electrode performance. MnO_2_ based electrodes are very popular for all mentioned characteristics with high catalytic effect at an appropriate potential for sensing the target analyte [17]. The aim of this work was to find a simple and cheap procedure for the determination of Vitamin B_2_ at low concentrations based on a manganese dioxide bulk-modified carbon paste electrode (MnO_2_/CPE).

## Experimental

### Chemicals

Boric acid, sodium hydroxide, acetic acid, phosphoric acid and manganese(IV) oxide (99.99 %, diameter approximately 5 μm), were purchased from Sigma–Aldrich (https://www.sigmaaldrich.com/) and used as received without any further purification. Calibration solutions were prepared from the stock solution (1 mM) by appropriate dilution with supporting electrolyte. Britton–Robinson buffer was prepared in usual way by mixing of 40 mM of all necessary components (phosphoric acid, acetic acid and boric acid). The pH of different Britton–Robinson buffer was adjusted with sodium hydroxide (0.2 M).

Working solutions of vitamin B_2_ (VB_2_) were freshly prepared on the day of the experiment by appropriate dilution with the supporting electrolyte. All other chemicals were of analytical reagent grade. Deionized water with a resistivity of 18 MΩ cm (Millipore Milli-Q system) was used for the preparation of all the solutions.

### Apparatus

Cyclic voltammetric (CV) measurements and differential pulse voltammetric (DPV) measurements were performed using an Autolab PGSTAT 302 N (http://www.metrohm.com/de-at) potentiostat/galvanostat controlled by corresponding software (Nova 1.10). The electrochemical cell (total volume of 10 mL) consisted of a glass vessel equipped with the Ag/AgCl (3 M KCl, Metrohm 6.0733.100) as a reference electrode, platinum wire as a auxiliary electrode and carbon paste as a working electrode. All of the pH values were measured using a pH meter (Orion, model 1230) with a combined electrode (glass-reference electrodes), which was calibrated weekly with standard buffer. All potentials given in the text are versus the Ag/AgCl reference electrode at room temperature.

### Preparation of a carbon electrode modified with manganese dioxide (MnO_2_/CPE)

Plain carbon paste was prepared by carefully hand mixing 380 μL of paraffin oil with 1 g of graphite powder in a mortar with a pestle. After standing overnight a portion of the resulting paste was packed into the end of a Teflon tube (an inner diameter 5 mm, outer diameter 10.15 mm) and the surface was polished using a PTFE plate or wet filter paper. The carbon paste was modified by adding 5 % (m/m) of MnO_2_ as received. The amount of modifier was selected according to our experience and previously described articles, where it is found that modification with 5 % of MnO_2_ gives best analytical response [11].

Whenever regeneration was required, a layer of the surface was removed and replaced by fresh paste. Electrical contact was made with a copper wire through the center of the tube.

### Procedures

Cyclic voltammetry with a scan rate of 0.1 V/s (if not stated otherwise) was used for characterizing the electrochemical behavior of the analyte at the unmodified and modified electrode surface. The investigated solutions were transferred into the voltammetric cell and the voltammograms (usually 5 cycles) were recorded in a potential range between −0.5 V and +0.5 V.

Differential pulse voltammetry with optimized parameters in the potential range from −0.5 V to +0.3 V (pulse amplitude of 0.12 V and pulse time of 0.04 s) was used for the quantification of VB_2_.

### Interference studies

Oxidation behavior of some possible interferences such as vitamins B_1_, B_6_ and B_12_, ascorbic acid and glucose, were tested in concentrations of 1 μM under optimized experimental conditions. The changes of the peak current of 1 μM VB_2_ were compared in the absence and in the presence of selected interferences_._ It was considered that tested compounds strongly interfere with the determination of riboflavin if gives signal changes more than ±10 %.

### Sample analysis

Vitamin B_2_ tablets (4.55 g) were dissolved in 10 mL of water and an aliquot (10 μL) was added to 10 mL of buffer at pH 2.0 and recorded by DPV under optimized experimental conditions. The concentration of VB_2_ was evaluated from calibration curve. All experiments were performed in triplicate.

## Results and discussion

### Electrochemical behavior of vitamin B_2_ on MnO_2_/CPE

**Fig. 1:**
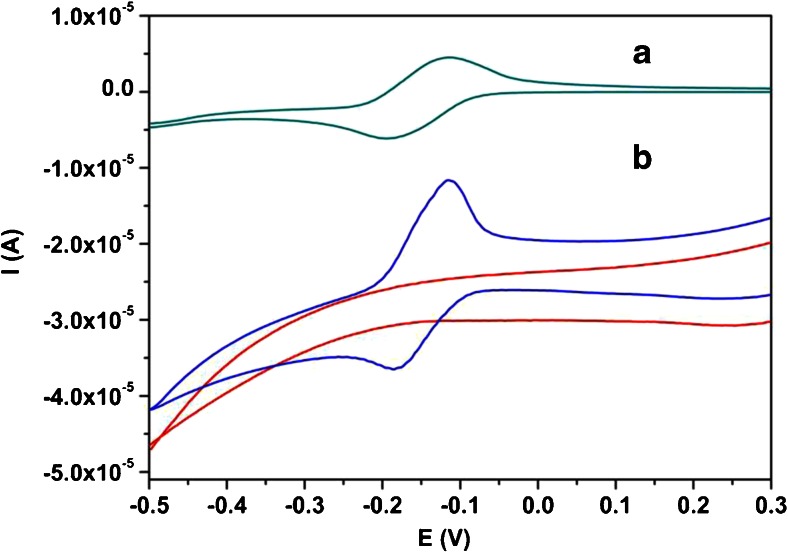
Cyclic voltammograms of 0.1 mM Vitamin B_2_ on A) CPE unmodified and B) MnO_2_/CPE in buffer at pH 2.0, scan rate of 0.1 V·s^−1^

Cyclic voltammetry was applied to study the electrochemical behavior of VB_2_ on a MnO_2_/CPE. All necessary factors influencing the current response of VB_2_ were carefully studied to explore the best conditions at which the best analytical performance was achieved. The electrochemical behavior of the MnO_2_/CPE towards VB_2_ was compared to the unmodified CPE electrode (Fig. 1).

The unmodified CPE (A) gave a small current response with well-defined oxidation peak at around −0.15 V and reduction peak at around −0.2 V. The modified electrode CPE /MnO_2_ (B) gives a well manifested oval-shape signal response at −0.15 V in the anodic direction. In the reverse scan also the reduction is observable at around −0.2 V indicating that the oxidation and reduction of the analyte during the reaction is electrochemically reversible. It is noted that the current corresponding to MnO_2_/CPE electrode is at about two time higher value when compared to the unmodified electrode. This is mainly attributed to the higher active surface area of MnO_2_ particles present on the surface of modified CPE electrodes. The current response obtained for the modified electrode approves the effect of MnO_2_ in the electrode structure.

### Effect of pH value of supporting electrolyte

Effect of pH on peak current and peak potential was investigated in the range from 2 to 6 using buffer. The peak current decreased considerably beyond pH 2.0. The peak potential of VB_2_ was shifted to more negative potentials linearly with increasing of the pH of supporting electrolyte. Based on these facts pH 2.0 was chosen for further experiments. When considering pH from 2 to 6 the peak potential shift to the more negative values occurs with the corresponding equation *Ep* (*V*) = *−0.052* × *pH* − *0.01272.*

**Fig. 2:**
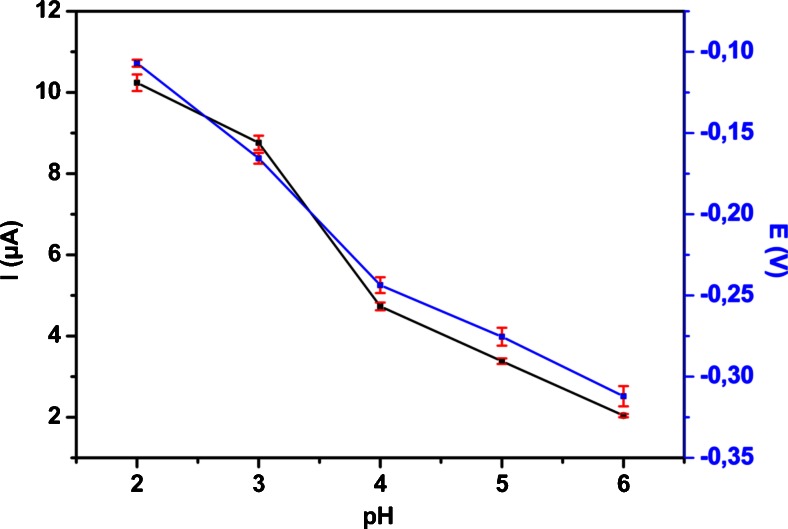
Effect of pH on the peak potential (■) and peak current (■) of 0.1 mM Vitamin B_2_ in buffer at pH 2.0 on MnO_2_/CPE using CV at scan rate of 0.1 V·s^−1^

The slope of 52 mV per pH unit is close to the ideal value of 59 mV which might indicate that the number of protons and electrons involved in the electrochemical reaction is in the ratio 1:1. In Fig. 2 the dependence of the peak current (Ip) and of the peak potential (Ep) on the pH of buffer is represented. Obtained proton/electron ratio is same as those previously described in the literature and in accordance with the oxidation reaction of riboflavin where two electrons and two protons are involved.

### Effect of scan rate

**Fig. 3:**
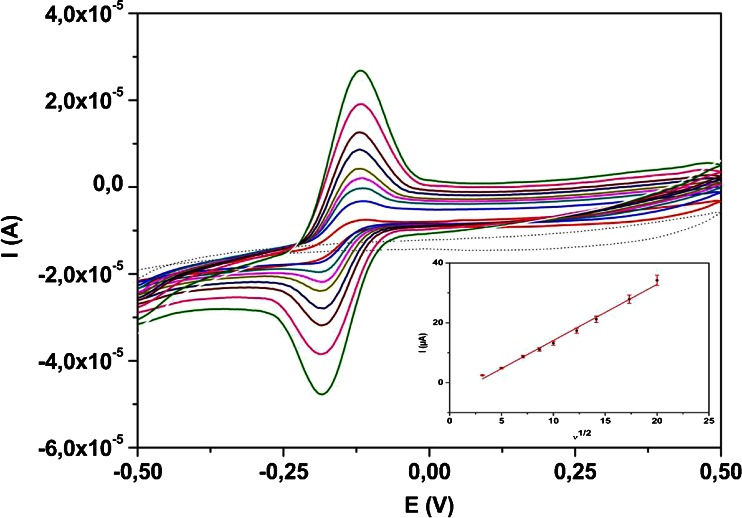
Cyclic voltammograms of 0.1 mM Vitamin B_2_ in buffer at pH 2.0 on MnO_2_/CPE at various scan rates from 0.01 V·s^−1^ to 0.5 V·s^−1^. The peak current (Ip) as a function of v^1/2^ for the oxidation peak of Vitamin B_2_ is shown in the inset

In order to study the nature of the electrochemical reaction of VB_2_ on MnO_2_/CPE the effect of different scan rate in the range from 0.01 V·s^−1^ to 0.5 V·s^−1^ on the peak current and peak potential was investigated by CV (Fig. 3) in buffer at pH 2.0. The peak current of VB_2_ increased practically linearly with square root of the scan rate indicating that the oxidation and reduction process on the electrode surface is controlled by diffusion rather than by adsorption. Inset of Fig. 3 the linear dependence can be expressed by the equation: *Ip* (μA) = *1.8760 x v*^*1*/*2*^ (*mV*·*s*^*−1*^) – *4.662* (R^2^ = 0.9927). Increase of the scan rate does not cause significant changes in redox peak potentials (ΔEp ~70 mV). These results indicate reversible process for the nature of electrochemical reaction.

### Optimization of DPV parameters

For the quantitative determination of VB_2,_ DPV was used as a suitable electroanalytical technique due to the low background currents and low detection limits. The parameters for DPV such as pulse amplitude and pulse time were optimized to find the best experimental setup for the quantification of VB_2_. The optimization was carried out in previously selected buffer at pH 2.0 with the concentration of 0.1 mM VB_2_. During this optimization procedure one investigated parameter was varied while the others were kept fixed. When the pulse time was varied from 0.01 to 0.1 s, the peak current increased up to value of 0.04 s and with further increase of the pulse time obtained current was decreasing. The most appropriate peak currents was observed at 0.04 s. Varying the pulse amplitude in the range of 0.01–0.35 V the peak currents increased with concomitant broadening of the peaks; finally a value of 0.12 V of pulse amplitude was chosen which was found to be most appropriate with respect to the current response and peak shape of VB_2_. All other experiments such as interference studies, calibration curve and sample analysis were carried out under these optimized parameters.

### Analytical performance

**Fig. 4:**
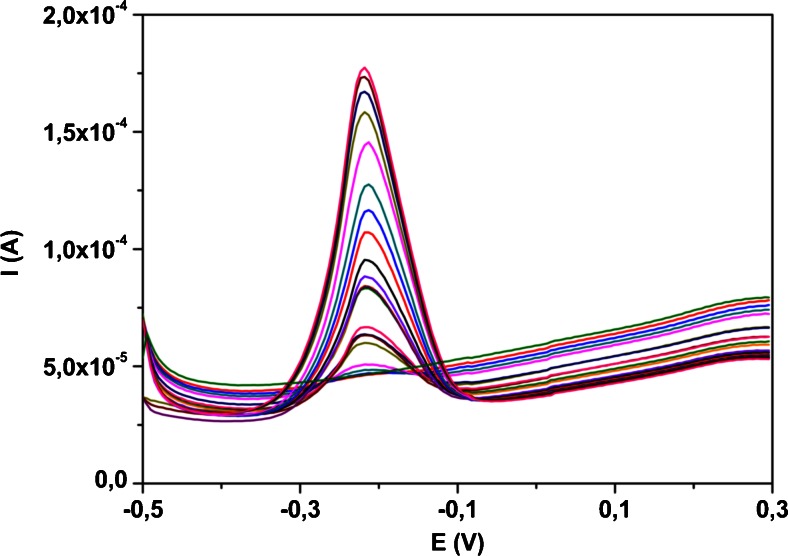
DP voltammograms for different concentrations of Vitamin B_2_ from 0.02 to 9 μM in buffer at pH 2.0 on MnO_2_/CPE at optimized DPV parameters

Calibration curve for determination of VB_2_ on MnO_2_/CPE was obtained using DPV under the optimized experimental conditions and was constructed by plotting estimated oxidation peak current versus known VB_2_ concentrations. Figure 4 shows a typical DP voltammograms obtained for different concentrations of VB_2_. The obtained currents were linear with logarithm of concentration in the range from 0.02 to 9 μM.

The graph shows a dynamic range for concentrations from 0.02 to 9 μM, with a corresponding linear equation: *I* (μA) = *55.51723 x log*_*C*_ μM + *92.31389*. Correlation coefficient of R^2^ is equal to 0.9976. The detection limit (3σ_c=0.06μM_/slope) was estimated as 15 nM. The repeatability (*n* = 4 measurements, c = 0.06 μM VB_2_) was calculated as ±2 %. RSD, and the reproducibility of the electrode preparation, based on measurements of the 0.06 μM of VB_2_ with four separately prepared electrodes, was estimated to be ±3 %, which improves our statement that this electrode can be satisfactory replacement for commercial electrodes.

This sensor offers low detection limit, wide linear range with a good sensitivity and reproducibility and in comparison with previously reported data this sensor possess comparable or better characteristics for the quantification of VB_2_.

Table 1 shows a comparison of the MnO_2_/CPE with other electrodes described recently in the literature. The method presented in this work has a comparable or better performance with wide linear range and low detection limit. The advantages of our method are based on simplicity of the electrode preparation.

**Table 1:** Recently reported electrodes for the determination of riboflavin

Electrode	Modifier	Method	Limit of Detection	Linear Range	Ref.
Gold Electrode	homoadenine single-stranded DNA/molybdenum disulfide–graphene nanocomposite	DPV	20 nM	0.025–2.25 μM	[18]
Carbon-paste electrode	zeolite	CV	0.71 μM	1.7–34 μM	[19]
Copper	Bi-film	SWAdSV	100 nM	0.3–0.8 μM and 1.0–9.0 μM	[2]
Pencil graphite electrode	DNA	DPV	0.9 μM	1–186 μM	[20]
Carbon paste	MnO_2_	DPV	15 nM	0.02 to 9 μM	This work

### Interference studies

**Fig. 5:**
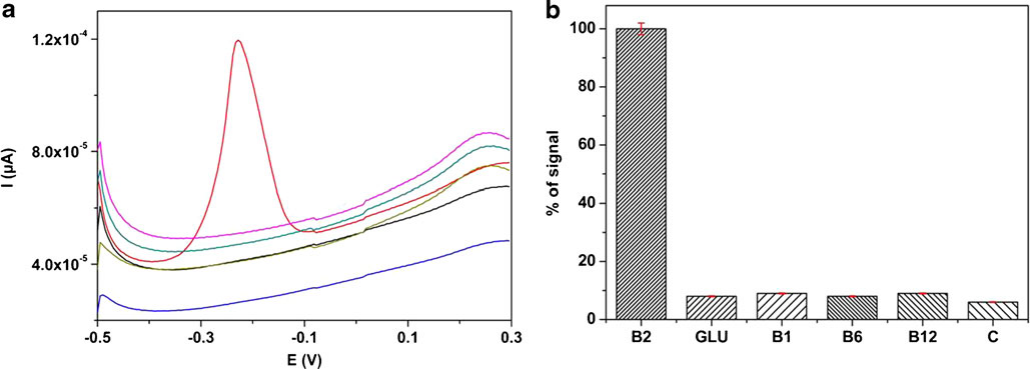
**a** DPVs of all tested compounds in concentration of 1 μM in absence of VB_2_ and **b** Signals of tested compounds in the presence of 1 μM VB_2_, expressed as relative signals of VB_2_ on MnO_2_/CPE in buffer at pH 2.0 at scan rate of 0.1 V·s^−1^

In order to evaluate the selectivity of the method toward VB_2_, the effect of possible interfering agents was investigated under optimized conditions. Some possible interfering compounds were tested, such as vitamin B_1_, B_6_, B_12_, ascorbic acid and glucose. These compounds in concentration of 1 μM, in absence of VB_2_, practically do not provide electrochemical activity in the tested potential range (Fig. 5 a). The presence of these interferences in same concentration level as VB_2_ (1 μM) do not causes changes in the peak current obtained for VB_2_ (Fig. 5 b). Hydrogen peroxide, dopamine and uric acid can be expected as possible interferences in the human body fluid samples (urine and blood serum). According previously published data [11, 21, 22] in strongly acidic media these compounds provides oxidation peaks at higher potentials compared to VB_2_ (~ −0.2 V). Based on these results this method has a good selectivity for the electrochemical determination of VB_2._

### Analytical application

**Fig. 6:**
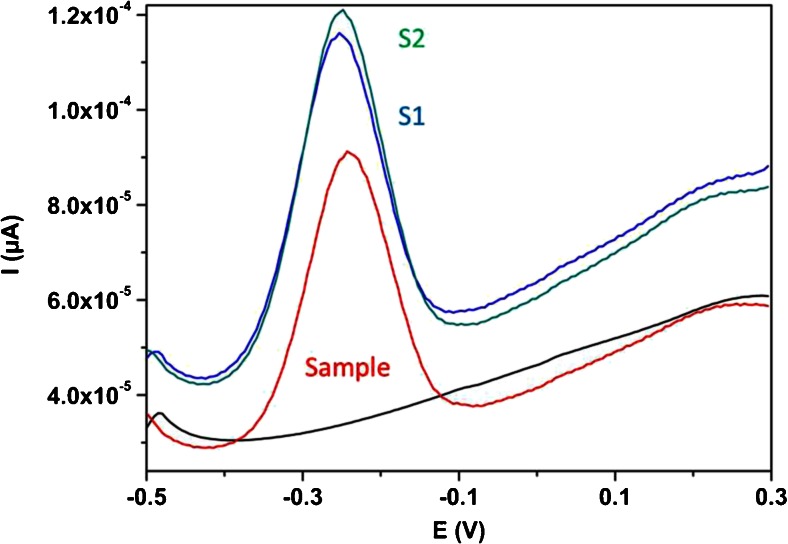
DP voltammograms obtained for determination of Vitamin B_2_ (S1-S2 standard addition 1 and 2) at MnO_2_/CPE electrode, in buffer at pH 2.0, under optimized experimental parameters

To test the practical applicability of the method it was applied for the analysis of VB_2_ content in the pharmaceutical formulations. The content of VB_2_ was determined from the calibration curve by optimized DPV method. The samples were prepared as it is previously described. The mean value of the concentration obtained by the calibration curve as 0.24 μM corresponds quite well to the labeled value of the commercial pharmaceutical formulation 0.26 μM. Standard addition of different amount of VB_2_ caused current increments at the sample potential (Fig. 6) which allows the evaluation of the recovery values.

The found values are in good agreement with added amount of VB_2_ and the accuracy was evaluated with recovery experiments (Table 2). The results are confirming that the sensor was applied for the determination of the concentration of VB_2_ in pharmaceutical formulations.

**Table 2:** Results for determination of VB_2_ in pharmaceutical formulations

Sample/Tablet	Labeled/μM	Found/μM	Added(S1)/μM	Found(S1)/μM	Recovery/%	Added(S2)/μM	Found(S2)/μM	Recovery/%
	0.24	0.26	0.10	0.35	97	0.2	0.53	95

## Conclusions

Carbon paste electrode modified with manganese dioxide was described for the determination of the vitamin B_2_ by differential pulse voltammetry. The observed results showed that incorporating of MnO_2_ in the structure of carbon paste electrode increases its affinity towards determination of riboflavin with a good reproducibility and very low detection limit. Based on the simplicity of the electrode preparation, its sensitivity and selectivity we propose simple, inexpensive electrochemical sensor which can be used for application on the field of riboflavin analysis.
